# A hygienic intervention program to decrease post-operative wound infections following CABG

**DOI:** 10.1186/2047-2994-4-S1-O32

**Published:** 2015-06-16

**Authors:** B Lytsy, RPF Lindblom, U Ransjö, CL Swenne

**Affiliations:** 1 Department of Clinical Microbiology and Infection Control, Uppsala University, Uppsala, Sweden; 2 Department of Thoracic Surgery and Anesthesiology, Uppsala University Hospital, Uppsala University, Uppsala, Sweden; 3 Department of Public Health and Caring Sciences, Uppsala University, Uppsala, Sweden

## Introduction

The department of Cardiothoracic surgery at Uppsala University Hospital has 25 beds in 1-4 patient rooms and an operating suite consisting of 5 operating rooms with ultraclean air. Around 700 open heart (250 isolated Coronary artery by-pass grafting, CABG) operations are performed annually. In 2009, the numbers of deep sternal wound infections (DSWI) increased to unacceptable rates despite existing hygienic guidelines.

## Objectives

To show how root cause analysis followed by quality improvement interventions reduced the rate of DSWI after CABG surgery.

## Methods

Only isolated CABG patients requiring surgical revision due to DSWI were included.Swabs and tissue biopsies were taken during surgical revision and analysed with standard methods. DSWI were registered prospectively according to CDC definitions. A root cause analysis for infection was performed Sep 2009-April 2010. Interventions based on results of the root cause analysis and on nationally recommended practices were concluded in April 2010, and thought to have taken full effect by July 1, 2010. Air was actively sampled at ≤ 0.5 m from the sternal incision.

## Results

DSSI incidence rates per CABG operations decreased from 5.1% pre- to 0.9% post- intervention. Wound cultures pre-intervention grew *S. aureus* 27.1% and CoNS 47.1%, post-intervention *S. aureus* 23.1% and CoNS 30.8%. Air counts did not exceed 5 cfu/m^3^.

## Conclusion

Good facilities and ventilation in the operating room cannot guarantee low infection rates. Guidelines are necessary, but strong leadership among all professions is needed to ensure compliance to guidelines.

## Disclosure of interest

None declared.

